# Changes in Structure, Interstitial Cajal-like Cells and Apoptosis of Smooth Muscle Cells in Congenital Ureteropelvic Junction Obstruction

**Published:** 2014-01-31

**Authors:** Mitra Mehrazma, Parin Tanzifi, Naser Rakhshani

**Affiliations:** 1 Oncopathology Research Center,; 2 Department of Pathology; 3 Children’s Medical Center, Pediatrics Center of Excellence, Tehran University of Medical Sciences, Tehran, Iran; 4 Firouzgar Hospital, Iran University of medical sciences

**Keywords:** Uretero-Pelvic Junction Stenosis, Image Analysis, Immunohistochemistry, Interstitial Cell of Cajal-Like Cells

## Abstract

***Objective:*** The goal of this study is to evaluate some structural changes in muscular, collagenous and neural components as well as expression of Cajal-like cells and apoptosis of smooth muscle cells in congenital ureteropelvic junction obstruction (UPJO).

***Methods:*** Tissue specimens were obtained from 25 patients with UPJO and compared with normal ureteropelvic junction regions of 19 autopsies. In paraffin embedded sections the amount of Cajal-like cells, density of nerve fibers and smooth muscle cell apoptosis (using immunohistochemical staining) were determined. Collagen deposition and muscular components were stained by Trichrome-Masson staining and evaluated by image analysis techniques. Arrangement of muscular bundles was also evaluated qualitatively.

***Findings*** ***:*** The number of Cajal-like cells was significantly lower in patients than in controls. The apoptotic score and mean number of nerve fibers were not statistically different for the two groups. Arrangement of muscular fibers was more irregular in patients than in controls (*P*<0.001). Collagen deposition was significantly higher in patients than in controls (*P*<0.001). The mean amount of muscular component was lower in patients than in normal ones. (*P*= 0.09)

***Conclusion:*** We found significant pathologic changes in congenital ureteropelvic junction obstruction such as decrease in Cajal-like cells, increase in collagen deposition and irregular arrangement of muscle fibers.

## Introduction

Ureteropelvic junction obstruction (UPJO) is the most common cause of congenital obstruction of urinary system with the prevalence of 1 in 1000 to 2000 neonates^[^^1^^-^^3^^]^. However, the mechanism of this obstruction has not been known well. This has attracted the attention of many investigators. It is now believed that the disease is multifactorial. It seems that the decrease in the number of smooth muscle cells, interstitial Cajal-like cells and nerve fibers in this region together with abnormal arrangement of smooth muscle cells and increase in collagen deposition have roles in the pathogenesis of the disease^[^^4^^]^. The number of studies on the role of apoptosis in smooth muscle cells is also limited and has led to different results^[^^5^^-^^8^^]^. 

 In the proximal parts of the urinary system, peristaltic contractions result in the outflow of urine from pelvis through the ureter to the bladder^[^^3^^]^. The outset of these contractions results from the spontaneous production of pulse potentials that start in proximal part of calyceal system and adjust by pacemaker tissue^[^^3^^]^.

 The interstitial cells of Cajal have been known as pacemakers for the contraction activity of gastrointestinal system. Some studies have been done on the role of Cajal-like cells as pacemakers in the upper parts of the urinary system^[^^2^^,^^3^^,^^5^^,^^6^^,^^9^^-^^15^^]^. 

 The goal of this study was to examine a series of pathologic changes occurring in congenital ureteropelvic junction obstruction. The results can help with the expression of new therapeutic modalities.

## Subjects and Methods

For the patient group, we used tissue specimens from ureteropelvic junction of 25 children with the mean age of 20 months (50 days to 8 years) with congenital ureteropelvic junction obstruction who underwent pyeloplasty. Prior to the surgery, their problem was confirmed by imaging study, and other causes of urinary obstruction were ruled out. Ureteropelvic junction of 19 matched autopsies with no obstruction in this region was used as control.

 Paraffin embedded blocks were first prepared from the specimens followed by slide preparation from each specimen and stained by H&E method and trichrome-masson staining. Immunohisto-chemical stained slides of S-100 (Polyclonal Rabbit Anti-Human, code N1573 Dako Cytomation, Denmark) for evaluation of nerve fibers, c-kit (Polyclonal Rabbit Anti-Human CD117, code A4502 Dako Cytomation, Denmark) for Cajal-like cells and caspase-3 (CPP32, clone JHM62, Novocastra Laboratories Ltd, Newcastle Upon Tyne, United Kingdom) for apoptotic smooth muscle cells were prepared according to the instructions of the factory in charge of the production. All slides were observed under light microscopic examination. Interstitial Cajal-like cells were counted in the randomly-selected 5 HPF in each group. Since mast cells are also immunoreactive for c-kit, only those cells with large vesicular nuclei, asteroid shape and small perinuclear cytoplasms having two or more slender cytoplasmic processes localized inbetween muscle fibers were counted. Nerve fibers were immunoreactive for S-100 and were counted in 5 HPF(s) too. Due to the cytoplasmic reaction of caspase 3 antibody, the apoptosis of smooth muscle cells was categorized as (<25%, 25-50%, 51-75%, >75%) with the intensity (0-3+) of immunoreaction to the marker. Collagen deposition has blue color in trichrome-masson staining and its amount was measured by computer analysis of 5 randomly selected HPFs in two groups. Muscular component is red in trichrome-masson staining and was quantitatively evaluated like collagen deposition. In image analysis, areas other than ureteral wall were not considered in measurement. Arrangement of muscle fibers was compared qualitatively between the two groups.

 In order to perform statistical analysis, the software SPSS-15 was utilized. We used abundance and ratio for qualitative variables and mean and standard deviation for quantitative ones. The comparison of the means and ratios was done by t-test and χ^2^-test respectively. *P* values less than 0.05 were considered to be significant. 

## Findings

**Fig. 1:**
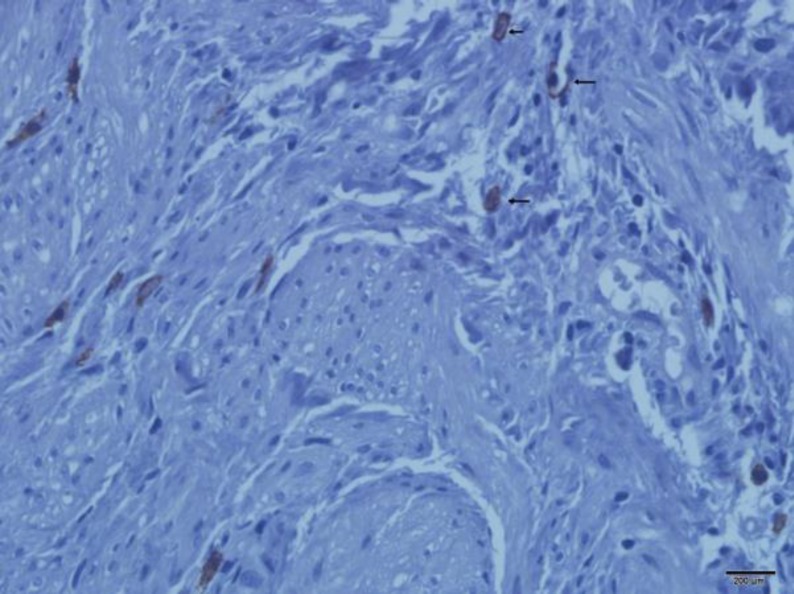
Cajal-like cells in a control specimen. Note to Cajal-like cells with 2 cytoplasmic processes in between muscle fibers in left side. There are also few mast cells in right side of the image (arrow). (x400)

The mean number of interstitial Cajal-like cells was 14.5±5.6 per 5 HPFs in patients and 32.8 ±11.9 per 5 HPFs in controls, which reveals significant difference between the two groups (*P*<0.001) (Fig. 1).

 The intensity of immunoreactivity of smooth muscle cells to caspase-3 was 0 in 7 (28%) patients, 1+ in 8 (32%), 2+ in 7 (28%) and 3+ in 3 (12%) patients. In the control group, 7 cases (36.8%) showed the intensity of 0, 7 (36.8%) 1+, 2 (10.5%) 2+, and 3 (15.9%) 3+ (*P=*0.2).

 From the standpoint of the extent of immunoreactivity of smooth muscle cells to caspase-3, it was <25% in 12 (48%) patients, 25%-50% in 5 (20%), 51%-75% in 4 (16%) and >75% in 4 (16%) cases. In the control group, this was <25% in 12 (63.1%) cases, 25%-50% in one (5.3%), 51%-75% in 4 (21%) and >75% in 2 (10.6%) cases (*P=*0.2).

 The mean score of immunoreaction to caspase-3 (which was calculated by multiple intensity extent of immunoreaction) in patients was 3.5±3.1 and in controls 2.8±2.5. 

**Fig. 2:**
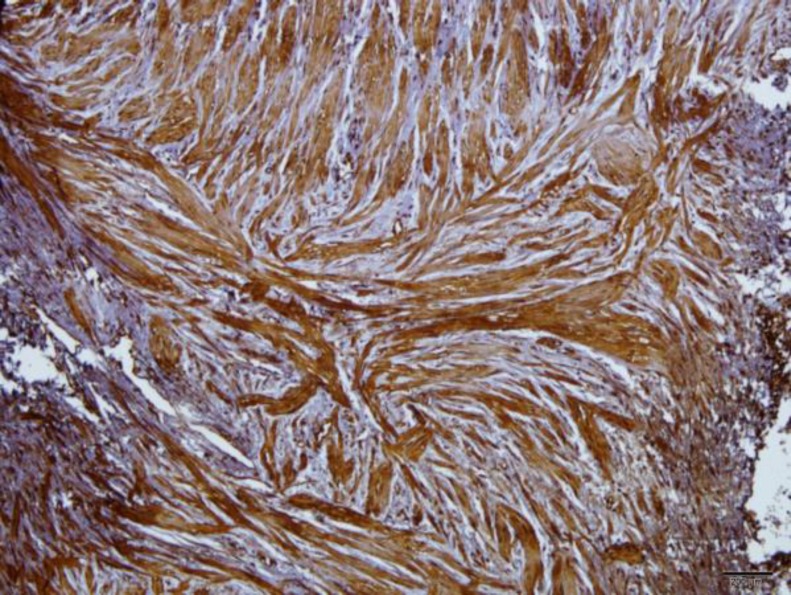
Immunoreaction of smooth muscle cells to caspase 3 antibody in a patient specimen.(intensified 3+) note also to the irregular arrangement of the muscle fibers.(×100)

There was no significant difference between the two groups (*P*=0.4)(Fig. 2).

 The mean number of nerve fibers in patients was 8.7±5.2 per 5 HPFs and 11.4±8.3 per 5 HPFs in controls, which shows no significant difference between the two groups (*P*=0.2).

 The arrangement of muscular fibers in 8 (32%) patients was regular and in 17 (68%) irregular compared to 17 (84.2%) regular and 2 (15.8%) irregular cases in the control group. Irregular arrangement refers to disorganization and sometimes haphazard arrangement of smooth muscle fibers with deposition of collagen fibers inbetween. Regular arrangement, however, refers to normal array of smooth muscle fibers in this region with inner circular and outer longitudinal layer of smooth muscle fibers. The difference was significant (*P*<0.001).

**Fig. 3:**
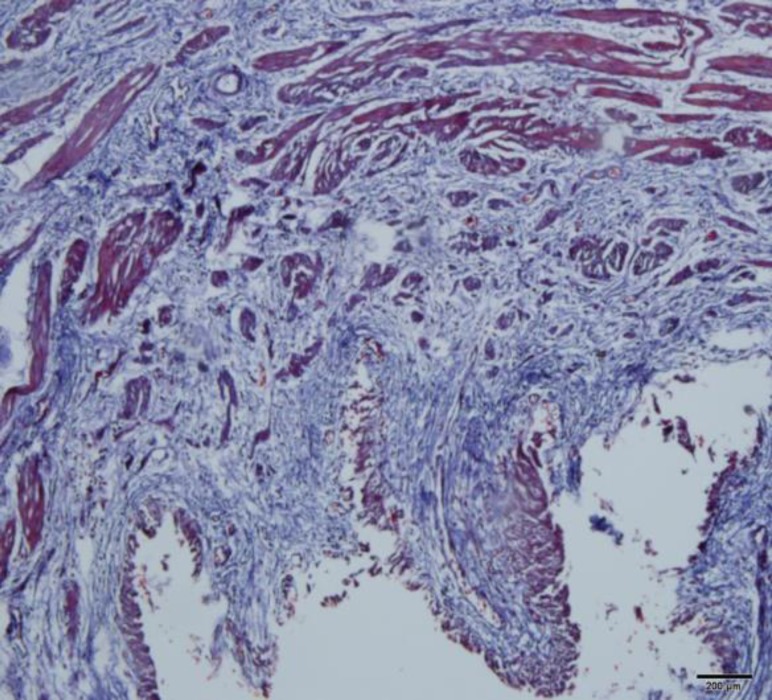
Increase in collagen deposition in a patient specimen. (Trichrome-Masson stain) (×100)

 The mean amount of collagen deposition in trichrome staining was 156000±82000 pX1^2^ per 5 HPFs in patients and 17800±6400 pX1^2^ per 5 HPFs in controls, this shows a significant difference between the two groups (*P*<0.001) (Fig. 3).

 The mean amount of muscular component in 5 randomly-selected HPFs in patients was 331200±94000 in comparison with 463100± 93000 in the controls which has no meaningful difference (*P*=0.09). Summary of the results are shown in Table 1.

## Discussion

The results of this study reveal that the mean number of interstitial Cajal-like cells in patients has significant statistical difference with that of the control group. Similar to the previous studies^[^^3^^,^^8^^,^^9^^]^, it shows that the decrease of these cells may have a role in the pathogenesis of the disease. This is in contrast with Koleda et al in which the number of interstitial Cajal-like cells sparse fields was lower in UPJO group than in controls^[^^10^^]^. They concluded that this issue would be probably due to the low mean age of their patients and appears as a primary compensatory effect.

**Table 1:** Comparison of results in patients with ureteropelvic junction obstruction and normal cases

**Variable**	**Patients**	**Controls**	***P.*** ** value**
**Mean number of Cajal-like cells per 5 HPFs**	**14.5 (5.6)**	**32.8 (11.9)**	**<0.001**
**Mean score of immunoreactivity of smooth muscle cells for caspase-3**	**3.5 (3.1)**	**2.8 (2.5)**	**0.4**
**Mean number of nerve fibers per 5 HPFs**	**8.7 (5.2)**	**11.4 (8.3)**	**0.2**
**Percentage of irregular arrangement of muscle fibers**	**68%**	**15.8%**	**<0.001**
**Mean amount of collagen deposition per 5 HPFs(pxl** ^2^ ** )**	**156000 (82000)**	**17800 (6400)**	**<0.001**
**Mean amount of muscular component per 5 HPFs(pxl** ^2^ ** )**	**331200 (94000)**	**463100 (93000)**	**0.09**

HPF: high power feild

Surprisingly, the mean age of our patients was also low (20 months) which compares well with theirs (2.3 years). We couldn`t rationalize this difference; however, it may be due to the limited number of their controls. Further studies with proper sample volume are needed in this regard to clear the subject.

 In this observation we did not find any significant difference for intensity, extent and total score of apoptotic smooth muscle cells between the groups of patients and controls. We found a few studies in the literature about smooth muscle cell apoptosis in UPJO. Kajbafzadeh et al^[^^1^^]^ showed that in UPJO there is an increase in smooth muscle cell apoptosis; nevertheless, they evaluated it with TUNEL assay, the method about which there are some criticisms in the literature^[^^16^^-^^18^^]^. Arena et al^[^^2^^]^

also observed the increase in the apoptosis of smooth muscle cells by using active caspase 3 and western blotting in UPJO specimens, but Özel et al^[^^5^^]^ couldn`t find any difference between patients and controls in number of apoptotic smooth muscle cells albeit they used Bax antibody for the detection of apoptosis. On the other hand, they found increase in the expression of Bcl2, an anti-apoptotic marker, they related this to the non-specific inflammation observed in their UPJO specimens. Here, two points should be considered: although there was no statistically significant difference, there were clinically some differences in intensity and overall apoptotic score in patients than in controls. Maybe with increase in the statistical population significant difference will appear. Because of the differences between the present data and a few studies on this subject more research is necessary to clarify the fact.

 The mean number of nerve fibers was lower in the patients group than in controls, but it was not statistically significant. With increase in the number of cases, it is probable to see significant difference between the two groups. Evidences from previous projects are more in favor of decrease in neural elements in patients suffering from UPJO^[^^1^^,^^4^^]^.

 We found a clear difference in the arrangement of muscular fibers between patients and controls that is almost irregular in the first group while being regular in the other. This shows that although in some patients decrease in the density of muscular component or apoptosis of smooth muscle cells is not obvious, consideration of the arrangement of muscular component can be helpful in confirmation of the disease. Besides, it may be due to the low mean age of the patients that we could not find meaningful results for the amount of muscular component, because with progression of the disease these structural changes probably will get more severe if they were actually present. Furthermore, we can conclude that irregular arrangement of muscular component may have a role in the pathogenesis of the congenital ureteropelvic junction obstruction, because it has not been known which of these changes occur earlier or later in comparison with each other in the pathologic process; more investigations are needed in this regard. 

 Our findings showed that the amount of collagen deposition in patients was more than in controls. This might be the result of atrophic changes due to cellular degeneration and substitution of extracellular matrix instead.

 There are considerable similarities between the present study and its predecessors. Özel et al found that in all specimens received from patients with UPJO, under light microscopic examination, there were nonspecific evidences of inflammation, epithelial proliferation, atrophy of smooth muscle fibers and fibrosis in the region of stenosis. Fibronectin, collagen type IV and laminin in the obstruction site were significantly more than in ureteropelvic regions of normal group. They concluded that significant increase of fibronectin, collagen type IV and laminin in the region of stenosis is one of the pathologic processes in congenital ureteropelvic junction obstruction^[^^5^^]^. Murakumo et al showed that muscle fibers became thinner in patients. Additionally, intercellular spaces became wider, collagen fibers increased significantly, and nerve fibers were less than in controls. They concluded that atrophic changes in muscle fibers and increase in fibrosis are important processes in the development of the disease^[^^4^^]^.

 We had some limitations in our research. Because of positive immunoreactivity of mast cells to c-kit antibody, it would have been better to check Cajal-like cells with another marker or staining method in addition. However, with respect to morphology of these two cell types and considering the location of Cajal-like cells (inner border of circular muscle layer), the probability of any mistake is low, specifically when we found significant difference between the two groups in immunoreaction to c-kit antibody. Furthermore, it is believed that in fibrotic areas number of mast cells is increased. So, with the assumption of happening the mistake being inevitable, the result would be in favor of increasing the number of c-kit positive cells in patient group (due to increase in mast cells) and therefore we expected this supposed mistake resulting in lower difference rate between the two groups.

## Conclusion

Our study reveals notable pathologic changes in the stenotic site of ureteropelvic junction in children suffering from congenital ureteropelvic junction obstruction. These changes include decrease in interstitial Cajal-like cells, irregular arrangement of muscle fibers and increase in collagen deposition.
